# Ochratoxin A Producing Species in the Genus *Penicillium*

**DOI:** 10.3390/toxins2051111

**Published:** 2010-05-14

**Authors:** Francisco Javier Cabañes, Maria Rosa Bragulat, Gemma Castellá

**Affiliations:** Veterinary Mycology Group, Department of Animal Health and Anatomy, Universitat Autònoma de Barcelona, Bellaterra, E-08193, Spain; Email: rosa.bragulat@uab.es (M.R.B.); gemma.castella@uab.es (G.C.)

**Keywords:** foods, ochratoxin A, *Penicillium*, taxonomy

## Abstract

Ochratoxin A (OTA) producing fungi are members of the genera *Aspergillus* and *Penicillium*. Nowadays, there are about 20 species accepted as OTA producers, which are distributed in three phylogenetically related but distinct groups of aspergilli of the subgenus *Circumdati* and only in two species of the subgenus *Penicillium*. At the moment, *P. verrucosum *and *P. nordicum* are the only OTA producing species accepted in the genus *Penicillium*. However, during the last century, OTA producers in this genus were classified as *P. viridicatum* for many years. At present, only some OTA producing species are known to be a potential source of OTA contamination of cereals and certain common foods and beverages such as bread, beer, coffee, dried fruits, grape juice and wine among others. *Penicillium verrucosum* is the major producer of OTA in cereals such as wheat and barley in temperate and cold climates. *Penicillium verrucosum *and *P. nordicum* can be recovered from some dry-cured meat products and some cheeses.

## 1. Introduction

Ochratoxin A (OTA) is a potent nephrotoxic mycotoxin that has been linked to kidney problems in both livestock and human populations. It has also carcinogenic, genotoxic and immunotoxic properties. Natural occurrence of OTA has been reported from temperate to tropical climates mainly on cereals and their products. However, it is also found in a variety of common foods and beverages, including bread, beer, chocolate, coffee, dried fruits, grape juice, pork, poultry and wine, among others [1]. In a recent study about the health risk assessment of OTA in a market economy [2], the major food contributors of OTA for children were wheat based foods followed by oats, rice and raisins; beer, coffee and wine also contributed to total OTA exposure in older individuals. The presence of OTA in blood from healthy humans confirms a continuous worldwide exposure [3]. In Europe, OTA maximum levels have been established for most of the above mentioned foodstuffs [4], some spices and liquorice [5] and also some guidance values for this mycotoxin have been also recommended for cereals, cereal products intended for animal feed and complete and complementary feedingstuffs for pigs and poultry [6].

Some species of the genera *Penicillium* and *Aspergillus* are known to form OTA, but few of them are known to contaminate foods with this mycotoxin. OTA contamination of food and feeds was until recently believed to be produced only by *A. ochraceus* and by *P. verrucosum*, which affect mainly dried stored foods and cereals respectively, in different regions of the world. However, some recent surveys have clearly shown that certain species belonging to the black aspergilli, including the *A. niger *aggregate and *A. carbonarius*, are sources of OTA in food commodities such as wine, grapes and dried vine fruits worldwide [7,8,9,10,11,12,13,14,15,16,17,18,19]. Recent studies also indicated that in addition to these species, *A. westerdijkiae*, *A. steynii *and* A. ochraceus *are responsible for the formation of OTA in coffee [20,21].

Species included in some of these taxa are difficult to distinguish from each other and molecular methods are usually necessary to identify them. For some of them, taxonomy is not fully resolved, as the number of accepted species depends on the methodology used. So far there has not been complete agreement between phenotypical and molecular data. In part for these reasons, a larger number of species have been cited incorrectly as OTA producers. This is also due mainly to the use of unsuitable analytical techniques in determining OTA production and misidentification of the fungal isolates tested [22]. A set of recommendations have been recently published to avoid incorrect reporting of fungal species producing particular mycotoxins [23]. The taxonomy of the OTA producing species in the genus *Aspergillus* and their potential for mycotoxin production in different foods have been reviewed in different papers [24,25,26,27,28,29,30]. A new subgeneric classification based on phylogenetic analysis of multilocus sequence data has been recently proposed [31]. 

In this paper, an overview of the current taxonomy of OTA producing species in the genus *Penicillium* arising from the most relevant approaches published in this field is presented. This review pays special attention to the natural occurrence of these species on food commodities where they are potential sources of OTA contamination. Emphasis has been placed on literature published within this decade, but prior noteworthy review papers and seminal works are included.

## 2. OTA Producing Species in the Genus Penicillium

### 2.1. Taxonomy

*Penicillium* taxonomy is not easy for the inexperienced, and compared to *Aspergillus* it is a more diverse genus, in terms of numbers of species and range of habitats [32]. At present, *P. verrucosum *and *P. nordicum* are the only OTA producers known and accepted in this genus, despite some reports on OTA production by other species [33,34]. *Penicilliumcasei* and *P. mediolanense* are synonyms for *P. verrucosum* and *P. nordicum*, respectively [34]. Nevertheless, different examples of incorrect citations of some *Penicillium *spp. producing OTA (e.g., *P. cyclopium*,* P. viridicatum*, *P. chrysogenum*) have been recently listed [23,34]. It is worth bearing in mind that in the last century, OTA producers in this genus were classified as *P. viridicatum* for many years. Main species concepts for *P. viridicatum*, *P. verrucosum* and *P. nordicum* are summarized in Table 1. 

**Table 1:** Main species concepts of OTA producing species in the genus *Penicillium*.

**References**	**Strains**		
	**OTA - and CIT -**	**OTA + and CIT +**	**OTA + and CIT -**
Frisvad & Samson., 2004 [33]	*P. viridicatum*	*P. verrucosum*	*P. nordicum*
			*P. nordicum *II OTA?
Larsen *et al.*, 2001 [35]	*P. viridicatum*	*P. verrucosum*	*P. nordicum*
Frisvad & Filtenborg, 1989 [36]	*P. viridicatum*	*P. verrucosum *chemotype II	*P. verrucosum *chemotype I
Pitt, 1987 [37]	*P. viridicatum*	*P. verrucosum *chemotype CIT	*P. verrucosum*
Pitt, 1979 [38]	*P. viridicatum*	*P. viridicatum*	*P. verrucosum*
Samson *et al.*, 1976 [39]	*P. verrucosum var. verrucosum*	*P. verrucosum var. verrucosum*	*P. verrucosum var. verrucosum*
Ciegler *et al.*, 1973 [40]	*P. viridicatum *I	*P. viridicatum *II	*P. viridicatum *III
Raper & Thom, 1949 [41]	*P. viridicatum*	*P. viridicatum*	*P. viridicatum*

OTA, ochratoxin A; CIT, citrinin; +, producing strains; - non producing strains.

**Figure 1:**
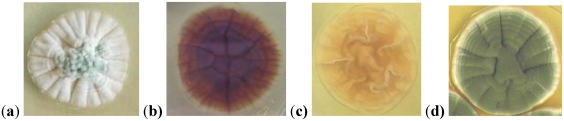
Colonies of some *Penicillium *spp. on Yeast Extract Sucrose agar after seven days of incubation at 25 °C (**a**) Colony pattern of *P. verrucosum* or *P. nordicum*; (**b**) *P. verrucosum* (reverse); (**c**)*P. nordicum* (reverse); (**d**) *P. viridicatum*.

*Penicillium verrucosum *and *P. nordicum* have common morphological characteristics such as very similar colony diameters on many culture media or rough stipes (Figure 1). These are slow growing species of the subgenus *Penicillium*, which is by far the most difficult taxonomically, both because there are numerous species and because apparent differences between species are small. Many species classified in this subgenus are morphologically similar, and identification using traditional morphological techniques remains difficult [32]. Recently, Frisvad and Samson [33] pointed out that a polyphasic approach, including a combination of DNA sequences, extrolite production and other phenotypical characters, is necessary to classify and identify species of *Penicillium* subgenus *Penicillium*. These authors keyed a total of 58 species in this subgenus. Many of these species are very common, being associated mainly with stored foods. The OTA producing species are placed in the series *Verrucosa* of the section *Viridicata* [33]. 

The series *Verrucosa* is monophyletic based on the phylogenetic analysis of partial β-tubulin sequences of strains representing all accepted species in this subgenus. This series includes two subclades, one consisting of the non OTA producing species *P. thymicola *and the other subclade consisting of strains of the two OTA producing species, *P. verrucosum* and two genetic groups of *P. nordicum* strains [42]. A similar genetic profile among these species was detected using *CO1* (mitochondrial cytochrome c oxidase 1) DNA barcoding [43]. In this study, *P. verrucosum* and three strains of *P. nordicum* had identical *CO1* barcodes, whereas four *P. nordicum *strains showed their own unique barcode. Currently, *P. viridicatum* is placed in the series *Viridicata* of the section *Viridicata* [33]. This series also includes other incorrectly cited OTA producing species such as *P. aurantiogriseum, P. cyclopium *or* P. polonicum*. Some years before, Larsen *et al.* [35] had described two distinct groups of OTA producing *Penicillium* strains based mainly on differences in secondary metabolite profiles. Some strains grouped with the ex-type culture of *P. nordicum* ATCC 44219 were classified as *P. nordicum*. Other strains grouped with the type culture of *P. verrucosum* NRRL 965 were classified as *P. verrucosum*. 

**Figure 2:**
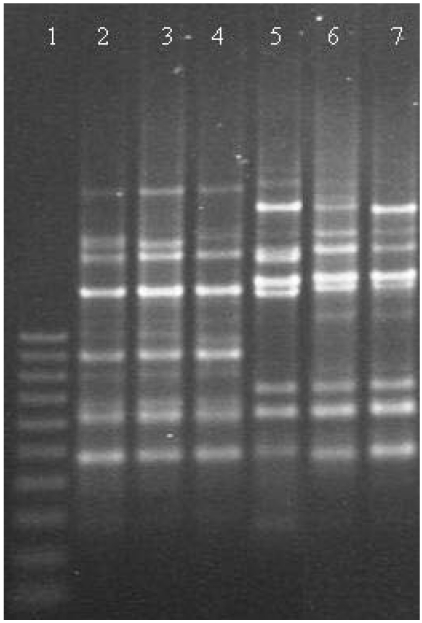
Agarose gel of representative RAPD banding patterns of *P. nordicum* and *P. verrucosum*. Lane 1, 100 bp ladder; lanes 2-4, *P. nordicum* patterns; lanes 5-7, *P.verrucosum* patterns.

These species are ecologically different. *P. nordicum* is generally recovered from meat and cheese products whereas *P. verrucosum* is recovered mainly from plant-derived material. Besides, most of the isolates of this latter species have a characteristic dark brown reverse color on Yeast Extract Sucrose agar (YES), whereas almost all the *P. nordicum* strains show a pale, creamy or dull yellow reverse color in this culture medium [33,35]. The colony pattern of *P. viridicatum *is different on YES (Figure 1). Among other differences, Frisvad and Samson [33] considered *P. verrucosum* among the species always negative (no reaction) or occasionally producing a yellow reaction for the Ehrlich test and *P. nordicum* among the species with a yellow reaction. These colored reactions are related to the production of some alkaloids. Castellá *et al* [44] confirmed these two groups of OTA producing strains in the genus *Penicillium* by Random Amplified Polymorphic DNA (RAPD) (Figure 2) and Amplified Fragment Length Polymorphism (AFLP) analyses, which are useful to differentiate these two species. However, the analysis of the ITS-5.8S rRNA gene sequences of these strains was not able to discriminate these two groups, indicating a close phylogenetic relationship between *P. verrucosum* and *P. nordicum*. 

### 2.2. Occurrence and Significance

*Penicillum verrucosum *is an important ochratoxigenic species because it is the major producer of OTA in cereals such as wheat, barley, oats and rye, in temperate and cold climates. This species is the main source of OTA contamination in cereals associated to the porcine and avian nephropathy detected in temperate and cold countries such as Denmark, Sweden, Canada or the United States [45,46]. An association of OTA with the human Balkan endemic nephropathy also has been suggested, but to date the etiology of this disease remains unresolved and controversial [47,48]. Contamination of animal feeds with OTA may result in the presence of residues in edible offal and blood products, whereas the OTA contamination in meat, milk and eggs is negligible. However, higher concentrations of OTA may occur in certain local specialties such as blood puddings and sausages prepared with pig blood serum [49]. At present, maximum levels for OTA in meat and meat products are not established in the European Community. However, the consideration of setting a maximum level for OTA for edible offal and blood products is under discussion. In Denmark, since 1978, the contamination of pig meat with OTA has been assessed indirectly by the inspection of pigs’ kidneys for the presence of macroscopic lesions of porcine nephropathy [50]. Nephritis is a common cause of condemnation of pig kidneys in Great Britain, but there are few studies of OTA in cases of porcine nephropathy identified at slaughter in other countries [51]. In France, the first national monitoring program showed that pigs are clearly exposed to OTA and monitoring of pork products and of feed for swine is necessary. Swine, like poultry, are exposed to OTA through their feed, which is composed of cereals such as barley, maize, oats and wheat that are susceptible to contamination by this mycotoxin [52]. In a recent study [53], more than 80% of the combine harvested samples (including rye, barley and wheat, among others) contained *P. verrucosum*, showing that much grain was contaminated prior to drying and storage. These authors pointed out that the early contamination with this species is a latent risk of OTA production if the grain is not handled properly after harvest.

It is well known that *P. verrucosum* is much more frequently found on cereals in countries where they occasionally have OTA problems as in the North European countries compared with South Europe where levels of OTA generally seem to be lower or they are not detected [54]. Recently, the occurrence of *P. verrucosum* on feedstuff and retail wheat flours purchased in the Spanish market was determined [55,56]. This species was the only OTA producing *Penicillium *species detected in these substrates. Although the occurrence and abundance of *P. verrucosum *producing species were moderately low in this study, these results confirmed the potential risk of OTA and CIT production in these products and the occurrence of *P. verrucosum* in South European countries.

*Penicillium nordicum* is normally present in the air and on the surface of hams in dry-cured ham manufacturing plants in Italy [57]. Recently, both species were also detected on the surface of sausages from northern Italy [58]. In this study, approximately 45% of these samples were positive for the presence of OTA. However, this toxin was not identified inside the dry meat. These authors [58] concluded that the presence of OTA on the surface of sausage constitutes a health risk when moulds are not removed from casings. *Penicillium verrucosum* has been also isolated from Speck [59] and Istrian dried ham [60].

In contrast, the OTA producing species *P. nordicum* and *P. verrucosum* were not isolated during some studies of the mycobiota of the processing areas of North European meat products, such as fermented sausage, liver pâté [61] and smoked dry-cured ham and dry-cured lamb leg [62]. Mould growth is not accepted on most types of North European meat products and is considered as both an economic and aesthetic problem for the producers. At the moment, neither of the two species, *P. nordicum* or *P. verrucosum,* have been cited from Spanish dry-cured meat products such as Iberian ham [63] or fermented meat sausages [64].

## 3. Conclusions

A high number of *Penicillium* species have been cited incorrectly as OTA producers. Species included in the subgenus *Penicillium* are difficult to distinguish from each other and molecular methods are usually necessary to identify them. On the other hand, different species concepts are used in the identification of these OTA producing species, causing confusion in the literature. At present, *P. verrucosum *and *P. nordicum* are the only OTA producers known and accepted in this genus. These species are phenotypically similar and phylogenetically closely related. However, they can be distinguished from each other mainly because they have different reverse color in YES agar and they produce different secondary metabolite profiles. They are also clearly differentiated by RAPD and AFLP analyses. On the other hand, the species are ecologically different. *Penicillium verrucosum* is the main source of OTA contamination in cereals and their products in cold and temperate climates. In contrast, *P*.* nordicum *is usually recovered from dry-cured meat products and cheese and it may be the cause of OTA contamination in these foods.
